# The Epitranscriptomic Mechanism of Metal Toxicity and Carcinogenesis

**DOI:** 10.3390/ijms231911830

**Published:** 2022-10-05

**Authors:** Chengfeng Yang, Zhishan Wang

**Affiliations:** Division of Cancer Biology, Department of Medicine, MetroHealth Medical Center, Case Western Reserve University School of Medicine, Cleveland, OH 44109, USA

**Keywords:** metals, arsenic, cadmium, chromium, epitranscriptome, epitranscriptomics, RNA modification, N6-methyladenosine (m6A), carcinogenesis

## Abstract

Metals are common toxic environmental pollutants. Acute or chronic exposure to metal pollutants causes severe adverse health effects in animals and humans, such as developmental retardation, abnormal metabolism, and disorders of cardiovascular, neurologic, respiratory, reproductive, and urologic systems. Moreover, several metals (arsenic, cadmium, chromium, and nickel) are classified as potent Group I carcinogens and cause various types of cancer in humans. Although the toxicity and carcinogenicity of metal pollutants are well recognized, the underlying mechanisms have not been clearly defined. The epitranscriptome includes all kinds of chemical modifications of all forms of RNA molecules inside a cell. Recent progresses in demonstrating the reversible pattern of RNA modifications and their roles in physiology and pathogenesis represent a breakthrough in the field of RNA biology and function study. The epitranscriptomic study is now an exciting emerging field in toxicology research. While few studies have been conducted so far to determine the epitranscriptomic effects of metal pollutants, they offer novel insights for understanding the mechanisms of metal toxicity and carcinogenesis. The goal of this review is to discuss recent studies on the epitranscriptomic effects of metals and propose some thoughts for future studies in the field.

## 1. Introduction

Toxic metals are common environmental pollutants and can be toxic to humans and animals even at low concentrations. Some toxic metals (arsenic, cadmium, chromium, and nickel) are classified as potent Group I carcinogens by the International Agency for Research on Cancer. The natural activities and widespread applications of metals in agriculture, industries, medical fields, and consumer products lead to their wide presence in the environment, representing a significant environmental health issue. Although toxicities and carcinogenicities of metal pollutants are well recognized, the underlying mechanisms are not well understood.

Previous studies have demonstrated that oxidative stress and genetic and epigenetic dysregulations could play important roles in metal toxicity and carcinogenesis, which have been discussed in recent reviews [1,2,3,4,5,6,7,8,9]. Over the past decade, an exciting new field in biological and biomedical research is epitranscriptomic study [8,9,10]. Emerging evidence shows that exposure to metals causes epitranscriptomic dysregulations, which may represent previously unrecognized novel mechanisms responsible for metal toxicity and carcinogenesis. The goals of this review are to briefly introduce epitranscriptomics and discuss recent studies on the epitranscriptomic effects of metals and their implications in metal toxicity and carcinogenesis.

## 2. Epitranscriptomics

### 2.1. A Brief History of Epitranscriptomics

Analogous to DNA methylation and nuclear core histone post-translational modifications (PTMs), RNA molecules are also chemically modified. The demonstrating of critical biological functions of RNA modifications led to the birth of the terms “RNA epigenetics”, “Epitranscriptome”, and “Epitranscriptomics” [11,12]. The epitranscriptome includes all types of chemical modifications of all kinds of RNA molecules (the transcriptome) inside a cell.

RNA chemical modifications were initially reported in the 1950s [13]. A large number of forms (more than 150 types) of RNA chemical modifications have now been characterized [14]. Although many types of RNA chemical modifications have been identified, the mechanisms and biological functions of RNA modifications have not been well understood. The low abundance of RNA modifications and the lack of sensitive techniques to detect and quantitate them for a long time are probably the major hurdles in dissecting the mechanism and functions of RNA modifications. Due to these limitations, the effects of environmental pollutants, especially toxic metal exposures on the epitranscriptome, have been rarely explored in the past 5 years.

Early RNA modification studies mainly focused on non-coding RNAs such as tRNAs (transfer ribonucleic acids) and rRNAs (ribosomal RNAs), as these two types of RNAs have a relatively high abundance of chemical modifications. In the 1970s, the first chemical modifications in messenger RNAs (mRNAs) were reported, and the subsequent discovery of the N6-methyladenosine (m6A) modification in mRNA changed the focus of RNA modification studies from tRNAs and rRNAs to mRNAs. It has been determined that m6A is the most abundant chemical modification in mRNAs, which occurs in around one to four m6As per every thousand adenosine nucleotides in mammalian mRNAs [15]. The methyltransferases that place m6A methylation in RNA molecules were discovered in 1990s [16,17]. The two m6A demethylases known as fat mass and obesity-associated protein (FTO) and alkB homologue 5 (ALKBH5) were discovered in 2011 and 2013 [18,19], respectively, which revealed the reversible and dynamic nature of RNA m6A modification. Moreover, these studies reporting the discoveries of two m6A demethylases also indicated that RNA m6A dynamic and reversible modification may have critical biological functions, which have significantly stimulated RNA m6A modification studies since then.

### 2.2. Modifiers and Regulators of the Epitranscriptome

The levels and functional outcomes of RNA modifications are determined by three groups of proteins that make the RNA chemical modification machinery: (i) writers, the proteins that write related chemical modifications onto RNA molecules; (ii) erasers, the proteins that erase chemical modifications from RNA molecules; (iii) readers, a variety of proteins that interact with RNA molecules bearing different types of chemical modifications and determine the functional outcomes of various RNA modifications [20].

**Figure 1:**
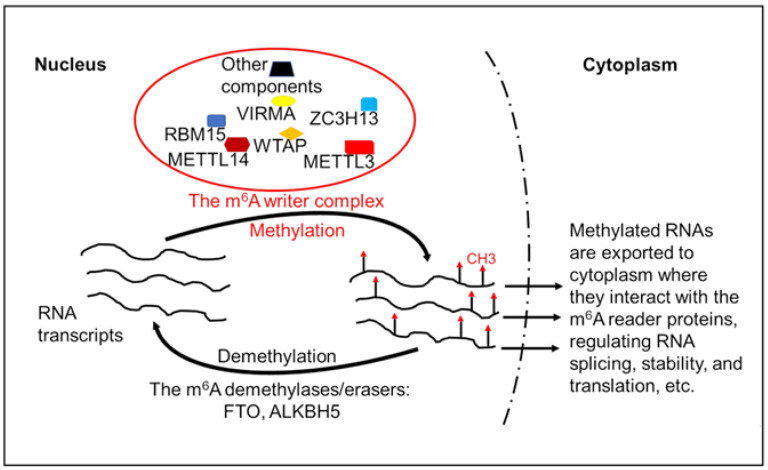
Regulation of RNA transcripts’ m6A modification. The RNA m6A modification is carried out by the m6A writer complex, which consists of multiple components including RNA methyltransferases and other proteins. The m6A-modified RNA transcripts could be demethylated by the m6A demethylase FTO or ALKBH5. The m6A-modifed RNA transcripts are exported to cytoplasm where they interact with the m6A reader proteins. The interactions between the m6A-modified RNA molecules and their reader proteins regulate RNA splicing, stability, and translation efficiency. METTL3, methyltransferase-like 3; METTL14, methyltransferase-like 14; WTAP, Wilms’ tumor 1-associated protein; VIRMA, Vir-like m6A methyltransferase-associated protein; ZC3H13, zinc finger CCCH domain-containing protein 13; RBM15, RNA-binding motif protein 15; FTO, fat mass and obesity-associated protein; ALKBH5, AlkB homologue 5.

Specifically, the level and function of eukaryotic mRNA m6A modification are modified and regulated by the following three groups of proteins: (i) The m6A writer is a multicomponent methyltransferase complex, which catalyzes the transfer of a methyl group from S-adenosylmethionine to the N-6 position of adenosine [20]. The key components in the m6A writer complex include methyltransferase like-3 (METTL3) and METTL14, Wilms’ tumor 1-associating protein (WTAP), and other proteins (Figure 1). Among these proteins, METTL3 is considered as the major catalytic methyltransferase. The other components of the m6A writer complex are also needed and work collaboratively with METTL3 to deposit the m6A modification to RNA molecules. (ii) As mentioned above, the erasers or the demethylases that clean the m6A from RNA transcripts are FTO and ALKBH5. (iii) The reader proteins that interact with m6A and mediate the biological functions of the m6A modification include the YTH domain-containing protein1-2 (YTHDC1-2) and YTH domain-containing family proteins1–3 (YTHDF1–3) [21,22,23].

### 2.3. Functions of Epitranscriptomic Regulations

Like DNA modifications and histone core protein PTMs, RNA modifications also affect gene expression. DNA modification and histone core protein PTMs affect gene expression mostly at transcriptional levels. In contrast, RNA modifications impact gene expression at post-transcriptional levels, translational, and post-translational levels. Studies have shown that RNA modifications could affect RNA splicing, trafficking, stability, and translation [23]. Therefore, RNA modifications may change gene expression through a variety of mechanisms, playing critical roles in normal physiology processes and pathogenesis as well [24,25].

The functional outcomes of RNA modifications are usually determined by the reader proteins that recognize and interact with various RNA modifications. The interactions between reader proteins and the modified RNAs may stabilize or destabilize RNA molecules and may increase or decrease mRNA translation efficiencies. For example, the interaction between YTH domain-containing family YTHDF2 and m6A-modified mRNAs reduces mRNA stability and increases mRNA degradation, causing down-regulations of gene expression [26,27]. In contrast, the interaction between YTH domain-containing family YTHDF1 and m6A-modified mRNAs increases mRNA stability and translation, leading to up-regulations of gene expression [26,27].

Considering the importance of RNA modifications in regulating gene expression, it is not surprising that RNA modifications are critically involved in normal development and pathological processes [24]. Studies have shown that RNA m6A modification regulators play important roles in the developmental processes of the brain, heart, skeletal muscle, and immune cell maturation [28,29,30,31]. For example, it has been found that nearly 25% of RNA transcripts in the healthy mouse and human heart display m6A modification [32], suggesting a critical role of RNA m6A modification in normal heart function. Indeed, significant changes in RNA m6A modification were observed during progression to heart failure [32].

Abnormal RNA modifications have been detected in a variety of diseases [24,33,34]. In particular, studies showed that RNA modification dysregulations could play important roles in cancer initiation, metastasis, prognosis, and responses to therapies [23,35,36,37,38,39,40,41,42]. Mechanistically, RNA modifications could affect the expression of genes involved in important oncogenic pathways such as the Wnt/β-catenin pathway, the PI3K-Akt-mTOR pathway, the JAK-STAT pathway, the MAPK pathway, the p53 pathway, the Hippo Pathway, etc. [23,43,44]. By regulating these critical oncogenic signaling pathways, RNA modifications could have significant impacts on cell survival, proliferation, apoptosis, malignant transformation, stem-cell-like properties, immune cell differentiation, maturation, and immune surveillance. Depending on the cellular context and signaling pathways involved, RNA modifications could promote or inhibit cancer development and progression [45,46,47].

## 3. Epitranscriptomic Mechanisms of Metal Toxicity and Carcinogenesis

### 3.1. Arsenic

Arsenic is naturally present at high levels in the groundwater of a number of regions or countries in the world. In addition, human activities and the widespread agricultural, industrial, and medicinal applications of arsenic release a large amount of arsenic into the environment. The U.S. Environmental Protection Agency (EPA) and the Agency for Toxic Substances and Disease Registry (ATSDR) list arsenic as a number 1 hazardous substance in waste sites on the U.S. National Priority List [48]. As a result, human exposure to arsenic is very common. General population exposure to arsenic occurs mostly by consuming arsenic-contaminated drinking water and food. Inorganic arsenic is highly toxic, exhibiting carcinogenic and non-carcinogenic effects. Chronic exposure to arsenic causes severe health issues, including but not limited to skin damages, cardiovascular disease, diabetes, neurological disfunctions, and multiple types of cancer [49,50,51]. In addition, studies showed that in utero and early childhood exposure has significant negative impacts on cognitive development [52]. While the toxic effects of chronic arsenic exposure are well recognized, the underlying mechanism has not been well understood. Previous studies on the mechanisms of arsenic toxicity and carcinogenesis have focused on oxidative stress, abnormal cell signaling, and genetic and epigenetic dysregulations, which were discussed in previous reviews [1,2,3,4,5,6,7].

#### 3.1.1. Epitranscriptomic Mechanism of Arsenic Toxicity

Bai et al. used the PC12 cell line and C57BL/6J mice as in vitro and in vivo models to study the mechanism of neurological disorders caused by arsenite exposure via drinking water [53]. It was found that significant learning and memory impairment and anxiety-like behaviors were observed in adult male mice exposed to arsenite (0.5, 5, and 50 ppm) in drinking water for 6 months [53]. Mechanistic studies showed that chronic arsenite exposure caused a dopaminergic transmission deficit to reduce dopamine concentrations in the cerebral cortices of mice. Since a previous study showed that the m6A eraser FTO is present in dopamine neurons and controls dopaminergic neurotransmission [54], the authors explored the role of FTO in the arsenite-induced dopaminergic transmission deficit. Interestingly, it was found that arsenite exposure increased the levels of m6A in PC12 cells in mice, which was associated with decreased protein levels of FTO [53]. These findings suggest that dysregulated RNA m6A modification resulting from arsenite exposure-caused down-regulation of its demethylase FTO, which might be involved in the dopaminergic transmission deficit and neurological disorders. Further studies are needed to determine whether FTO transgenic mice display resistance or reduced sensitivity to chronic-arsenic-exposure-caused neurological disorders.

To study the hormesis effect of arsenic exposure on human keratinocyte (HaCaT) cells, Chen et al. exposed HaCaT cells to different concentrations of sodium arsenite (0.5, 1, 2, 5, 10, 20, and 40 µM) for 24 h [55]. It was found that lower levels of arsenite exposure (0.5, 1, 2 µM) increased cell viability and higher levels of arsenite exposure (5, 10, 20, 40 µM) reduced cell viability. It was also determined that exposure to arsenite increased the intracellular ROS levels at all concentrations (0.5, 1, 2, 6, 12 µM) in a dose-dependent manner compared with the control [55]. Moreover, higher exposure levels (6, 12 µM) displayed more significant effects and increased the intracellular ROS levels by 3.31- and 4.98-fold, respectively. To explore whether the arsenite-exposure-caused cytotoxicity and oxidative stress are associated with any changes in the levels of RNA m6A modification, the authors detected the levels of m6A modification on total RNA of HaCaT cells [55]. Interestingly, all arsenite treatment groups had significantly higher levels of m6A modification than the control untreated group. However, the m6A levels peaked at the group of 2 µM treatment and declined at the groups of higher arsenite concentration treatments (6, 12 µM). Further analysis showed that the expression levels of m6A eraser FTO were decreased by the low concentrations (1, 2 μM) of arsenite exposure and were increased by the high concentrations of (6, 12 μM) of arsenite exposure [55]. In contrast, lower arsenite (1, 2 μM) exposure increased expression levels of several components (METTL3, METTL14, and WTAP) in the m6A writer complex, while high concentrations of arsenite exposure reduced the expression levels of METTL3, METTL14, and WTAP [55]. These findings showed that higher concentrations of arsenite-exposure-caused cell death are associated with the excess production of ROS and reduced levels of m6A modification.

Zhao et al. further determined the relationship between the levels of ROS and m6A modification in HaCaT cells exposed to different concentrations of arsenite [56]. It was found that treatment with an antioxidant N-acetylcysteine (NAC) reduced ROS levels and increased the viability of cells exposed to higher concentrations (5, 10, 15 μM) of arsenite, indicating that increased ROS levels contributed to higher levels of arsenite-exposure-caused cell death. It was also determined that only 15 μM of arsenite treatment significantly increased m6A levels in HaCat cells, the antioxidant NAC treatment significantly reduced m6A levels in cells exposed to 15 μM of arsenite. Further analysis showed that NAC treatment greatly reduced arsenite-exposure-caused up-regulation of METTL14 and WTAP, suggesting that high levels of arsenite exposure may increase RNA m6A modification levels by high levels of ROS-mediated up-regulation of METTL14 and WTAP expressions.

Arsenic-exposure-caused hepatic insulin resistance is an important early event in arsenic-induced metabolic disorders such as type 2 diabetes; however, the mechanism by which arsenic exposure induces hepatic insulin resistance remains largely unknown [57]. Qiu et al. exposed six-week-old C57BL/6 J male mice to 4 mg/L of As_2_O_3_ in drinking water for 6 weeks and established the early onset of hepatic insulin resistance in a mouse model as evidenced by the impaired glucose tolerance, insulin sensitivity, and insulin signaling [57]. It was determined that arsenic-induced NOD-like receptor protein 3 (NLRP3) inflammasome activation contributed to hepatic insulin resistance [57]. It was also found that the arsenite methyltransferase (AS3MT) interacts with and activates the NLRP3 inflammasome during arsenic-induced hepatic insulin resistance [57]. Additional mechanistic studies showed that arsenic exposure promoted METTL14 nuclear translocation, and knockdown of AS3MT reduced METL14 nuclear levels and total RNA m6A modification levels in arsenic-treated human fetal hepatocyte L-02 cells. Similarly, arsenic exposure also significantly increased mouse liver METL14 levels and total RNA m6A levels [57]. Further mechanistic studies showed that arsenic promoted NLRP3 expression and inflammasome activation via METTL14-dependent NLRP3 mRNA m6A modification [57]. Moreover, knockdown of METTL14 in L-02 cells reversed arsenic-treatment-caused insulin signaling impairment and increased glucose uptake. Together, these findings suggest that AS3MT-promoted and METTL14-dependent RNA m6A modification plays a critical role in arsenic-exposure-caused NLRP3 inflammasome activation and subsequent hepatic insulin resistance [57].

#### 3.1.2. Epitranscriptomic Mechanism of Arsenic Carcinogenesis

Cell culture and cell transformation models are widely used to study the mechanism of arsenic carcinogenicity. Gu et al. exposed human bronchial epithelial cells to 2.5 µM of sodium arsenite for 13 weeks to induce cell transformation, which was confirmed by the increased levels of cell proliferation, percentages of plate colony formation and soft agar clone formation, and resistance to apoptosis [58]. It was found that total RNA m6A modification levels were significantly increased in arsenite-transformed cells, which was accompanied by the increased protein levels of the m6A writer complex components METTL3, METTL14, and WTAP and reduced levels of m6A erasers FTO and ALKBH5 [58]. Moreover, knockdown of METTL3 in arsenite-transformed cells significantly reduced total RNA m6A levels and reversed their malignant phenotypes, as evidenced by the lower percentages of clone and colony formation as well as higher rates of apoptosis [58]. These findings indicate that chronic arsenic exposure increases total RNA m6A modification levels, which may play an important role in maintaining the transformed phenotypes of chronic arsenic-exposure-transformed human bronchial epithelial cells.

Similarly, Zhao et al. reported that total RNA m6A modification levels were also significantly increased in human keratinocyte HaCaT cells transformed by exposure to 1 μM arsenite for 5 months, which was accompanied by the increased protein levels of the m6A writer complex components METTL3 and METTL14 and reduced levels of m6A eraser FTO [59]. Knockdown of METTL3 in arsenite-transformed HaCaT cells significantly reduced their total RNA m6A levels and the transformed phenotypes [59]. Mechanistic studies showed that arsenite-transformed HaCaT cells exhibited reduced p53 activity and reduced levels of p53 phosphorylation, acetylation, and transactivation, with a high nucleus export rate of p53 [59]. In contrast, knockdown of the m6A writer complex key component METTL3 restored p53 activity in arsenite-transformed HaCaT cells [59]. It was further determined that increased m6A modification reduced the expression level of p53 activity positive regulator PRDM2 through the YTHDF2-promoted decay of PRDM2 mRNAs but increased the expression levels of p53 activity negative regulators YY1 and MDM2 through YTHDF1-stimulated translation of YY1 and MDM2 mRNAs [59]. These findings provide additional evidence supporting that up-regulation of m6A modification levels could play critical roles in maintaining the transformed phenotypes of chronic arsenic-exposure-transformed cells.

In contrast to studies discussed above, Cui et al. reported that total RNA m6A modification levels were significantly reduced in chronic arsenite-exposed HaCaT cells (0.1 µM for 28 weeks) [60], which was due to up-regulation of FTO since chronic arsenite exposure had moderate or no effect on m6A other regulators, including METTL3, METTL14, and ALKBH5. It was also found that FTO level was up-regulated while total RNA m6A levels were down-regulated in arsenic-associated human skin damages. Moreover, FTO knockout significantly reduced the growth of xenograft tumors produced by injection of arsenite-transformed HaCaT cells. Furthermore, epidermis-specific FTO deletion inhibited skin tumorigenesis induced by ultraviolet B light (UVB, wavelength between 280–315 nm) irradiation alone or the combination of UVB irradiation and arsenic, with a greater effect on the combination exposure [60]. Mechanistic studies revealed that NEDD4L is the m6A-modified gene target that plays an important role in arsenic tumorigenesis. It was further determined that arsenic exposure up-regulated FTO expression level by stabilizing the FTO protein through inhibiting p62-mediated selective autophagy [60]. Moreover, FTO up-regulation caused inhibition of autophagy, creating a positive feedback loop to maintain FTO accumulation [60]. Together, this convincing evidence demonstrated that FTO up-regulation caused down-regulation of mRNA m6A modifications, which plays an important role in arsenic carcinogenesis.

The important role of FTO up-regulation in arsenic mutagenesis has also been reported in another study. Gao et al. found that short-term arsenic (2 µM) treatment in lung cancer cells up-regulated the expression of APOBEC3B (A3B), an endogenous inducer of somatic mutations leading to chromosomal instability [61]. It was determined that up-regulation of A3B is required for arsenic-induced DNA damage and mutagenesis [61]. Mechanistic studies showed that arsenic treatment decreased the level of m6A modification near the stop codon of A3B mRNA, which increased the stability of A3B mRNA [61]. It was found that the protein level of FTO but not the other m6A modulators (METTL3, METTL1, METTL16, and ALKBH5), was up-regulated by arsenic treatment. Further mechanistic studies revealed that FTO reduced A3B mRNA m6A modification and decreased A3B mRNA stability in a YTHDF2-dependent mechanism. Moreover, it was determined that arsenic exposure also increased the expression of Apobec3 in mouse lung tissues in an FTO-dependent mechanism [61]. By analyzing lung tissue microarray data, it was found that expression levels of FTO and A3B were significantly higher in human lung cancer tissues than the adjacent normal lung tissues, and there was a significant positive correlation between FTO and A3B expression in lung cancer [61]. Further bioinformatics analysis showed that higher expression levels of FTO and A3B were associated with significantly worse overall survival in lung cancer patients [61]. Collectively, these findings suggest an important role of the FTO/m6A axis in arsenic-induced mutagenesis and lung cancer in general as well.

### 3.2. Cadmium

Cadmium is a naturally occurring toxic heavy metal and is usually produced as a byproduct in the processes of producing other metals. Due to its widespread industrial applications, a significant amount of cadmium has been released into the soil, water, and air. The U.S. EPA and the ATSDR list cadmium as one of the Top 10 Hazardous Substances in waste sites on the U.S. National Priority List [48]. In addition to occupational exposure to cadmium mainly through inhalation, general population exposure to cadmium could be commonly occurring through consuming cadmium-contaminated water, food, and air. Cadmium is highly toxic, having carcinogenic and non-carcinogenic effects. Due to its long biological half-life of 15–20 years, acute and chronic exposure to cadmium can cause multiple types of cancer and damage in multiple organ systems such as bone, the central nervous system, urinary and reproductive system, respiratory system, cardiovascular system, etc. [62,63]. Cadmium is a weak mutagen, displaying low genotoxicity especially at low doses. It is generally accepted that non-genotoxic effects such as epigenetic mechanisms play critical roles in cadmium toxicity and carcinogenicity, which are discussed in many reviews [1,2,3,4,5,6,7].

#### 3.2.1. Epitranscriptomic Mechanism of Cadmium Toxicity

To study the mechanism of cadmium reproductive toxicity, Ding et al. exposed bovine ovarian primary Granulosa cells to 5, 10, 15, 20, and 40 µM of CdCl_2_ for 24 h and found that all cadmium treatment significantly reduced cell viability and increased apoptosis [64]. Biochemical analysis showed that cadmium treatment induced oxidative stress, as evidenced by the reduced levels of antioxidants (CAT, SOD, and NQO1) and the increased levels of ROS production [64]. It was determined that cadmium treatment reduced FTO expression and increased m6A modification of MAX network transcriptional repressor (MNT) mRNA, leading to reduced expression of MNT. In contrast, overexpressing FTO reduced MNT mRNA modification and reversed cadmium treatment-caused down-regulation of MNT. Moreover, FTO overexpression in Granulosa cells suppressed cadmium-treatment-induced oxidative stress and apoptosis. It was further determined that siRNA knockdown of MNT reversed the protective effect of FTO overexpression on cadmium-treatment-induced apoptosis of Granulosa cells [64]. These findings suggest that abnormal m6A modification may play a critical role in cadmium reproductive toxicity.

In contrast, Sun et al. reported that cadmium treatment (CdSO_4_: 8–64 μM) induced oxidative stress and reduced the viability of human renal tubular epithelial cells (HK-2), which was accompanied by reduced expression of FTO but increased expression of METTL3 [65]. It was not determined whether cadmium treatment altered RNA m6A modification levels and whether dysregulated FTO and METTL3 expression played any role in cadmium-treatment-caused renal cell toxicity. Given the fact that cadmium treatment reduced FTO but increased METTL3 expression levels, it is highly likely that cadmium treatment may increase RNA m6A modification levels, leading to abnormal expressions of certain genes that may be involved in cadmium-caused renal injury.

Qu et al. found that cadmium treatment (CdSO_4_: 1, 2, 4 μM for 24 h) reduced viability and induced apoptosis of mouse pancreatic beta cells (NIT1), which was accompanied by the reduced total RNA m6A modification levels and the increased oxidative stress [66]. Western blot analysis showed that cadmium treatment reduced both FTO and METTL3 expression levels in NIT1 cells, with METTL3 down-regulation being more significant. However, the role of m6A modification dysregulation in cadmium-induced oxidative stress and pancreatic beta cell toxicity was not determined.

#### 3.2.2. Epitranscriptomic Mechanism of Cadmium Carcinogenesis

Wu et al. performed transcriptomic, epitranscriptomic, and proteomic analyses to investigate the mechanism of cadmium-induced human urothelial cell transformation [67]. A total of 9491 differentially expressed genes, 711 differentially expressed proteins, and 633 differentially m6A-modified genes were identified between the control cells and cadmium-transformed cells [67]. Further analysis revealed that 13 similarly dysregulated genes related to the onset or progression of cancer were shared between the m6A and proteomic data sets, suggesting that the m6A modification may be an important mechanism regulating the expressions of genes that are critically involved in cadmium-induced cell malignant transformation [67].

Li et al. reported that the protein level of the m6A eraser ALKBH5 was up-regulated, and the total mRNA m6A modification levels were significantly reduced in cadmium-transformed human bronchial epithelial BEAS-2B cells [68]. Knockdown of ALKBH5 in cadmium-transformed BEAS-2B cells not only up-regulated total mRNA m6A levels but also reduced their transformed phenotypes, as evidenced by the decreased proliferation, migration, invasion, and anchorage-independent growth [68]. The tumor suppressor gene PTEN was identified as a target gene of ALKBH5. It was determined that ALKBH5 reduced the m6A modification level of PTEN mRNA, leading to its instability and decrease in PTEN protein expression. Simultaneous knockdown of both ALKBH5 and PTEN reversed the inhibitory effect of ALKBH5 knockdown alone on the transformed phenotypes of cadmium-transformed cells [68]. These findings suggest that chronic cadmium exposure up-regulates ALKBH5 expression to reduce tumor suppressor gene PTEN mRNA m6A modification, down-regulating PTEN expression to promote cell transformation.

### 3.3. Chromium

Chromium (Cr) is a naturally occurring metal element and three main forms (valence) of Cr are: Cr(0), Cr(III), and Cr(VI). A large amount of hexavalent chromium [Cr(VI)] has been released into the environment due to its wide applications in many manufacturing processes. Cr(VI) is listed by the U.S. EPA and the ATSDR as one of the Top 20 Hazardous Substances in waste sites on the U.S. National Priority List [48]. Cr(VI) has carcinogenic and non-carcinogenic toxic effects. Earlier studies on the mechanism of Cr(VI) carcinogenicity mostly focused on its genotoxic effects [69,70,71,72,73,74]. More and more studies showed that Cr(VI) exposure is also capable of inducing non-genotoxic effects such as epigenetic effects, which are discussed in many reviews [3,6,7,75,76,77].

#### 3.3.1. Epitranscriptomic Mechanism of Chromium Toxicity

To determine whether Cr(VI) could exhibit its cytotoxic effect through dysregulating the RNA modifications, Chen et al. used the LC-ESI-MS/MS analytical method to investigate the effects of Cr(VI) exposure on 14 kinds of modifications in mRNA of HEK293T cells [78]. It was found that the modification of inosine is the only one significantly decreased in HEK293T cells exposed to 1 or 5 μM of Cr(VI) (K_2_CrO_4_) for 24 h, indicating that the Cr(VI) may affect the A-to-I (adenosine to inosine) editing in mRNAs [78]. Mechanistic studies revealed that the decrease in the level of inosine in mRNAs was due to the reduced expression of the adenosine deaminase acting on RNA (ADAR1) resulting from Cr(VI) exposure [78]. Since RNA A-to-I modification is widespread in living organisms and has important biological roles, these findings suggest that Cr(VI)-treatment-caused RNA A-to-I modification dysregulation may play an important role in the cytotoxicity of Cr(VI).

To study the mechanism of Cr(VI) reproductive toxicity, Lv et al. determined the effect of Cr(VI) treatment on the level of RNA m6A modification in mouse spermatogonial stem cells (SSCs) [79]. The authors first found that exposure of 8–10-week-old C57/BL6 male mice to Cr(VI) (Na_2_CrO_4_, 16.2 mg/kg b.w./day, ip) for 14 consecutive days damaged the reproductive system and SSCs [79]. Cell culture studies showed that exposure of mouse SSCs to 10 µM of Cr(VI) (Na_2_CrO_4_) for 1 h significantly reduced the total RNA m6A levels. It was further determined by using the m6A-IP-qPCR analysis that treatment with 10 μM Cr(VI) for 4 h reduced the m6A levels in mitochondrial fusion genes mitofusin 2 (Mfn2) and OPA1 mitochondrial dynamin-like GTPase (Opa1) and in mitophagy genes BCL2 interacting protein 3 (Bnip3) and BCL2 interacting protein 3-like (Nix) [79]. Mechanistically, it was determined that acute Cr(VI) exposure reduced the RNA m6A levels, probably through down-regulating the m6A writer METTL3 expression level [79]. Interestingly, the authors found that melatonin pretreatment alleviated Cr(VI)-induced damages to the male mouse reproductive system. Cell culture studies showed melatonin pretreatment attenuated Cr(VI)-induced mitochondrial disorders in SSCs, which was accompanied by the reversion of the Cr(VI)-caused decrease in RNA m6A modification levels in mitochondrial fusion genes Mfn2 and Opa1 and in mitophagy genes Bnip3 and Nix [79]. Moreover, it was further determined that METTL3 knockdown in mouse SSCs impaired the reversal effect of melatonin pretreatment on Cr(VI)-caused down-regulation of the m6A levels in mitochondrial genes and diminished the protective effect of melatonin pretreatment on Cr(VI)-caused mitochondrial damages [79]. These findings indicated that acute Cr(VI) exposure is capable of down-regulating METTL3 expression and reducing RNA m6A modification levels in mouse SSCs, which may play an important role in Cr(VI)-caused male reproductivity toxicity.

#### 3.3.2. Epitranscriptomic Mechanism of Chromium Carcinogenesis

We recently started exploring the epitranscriptomic mechanism of Cr(VI) carcinogenesis [80]. We first submitted the total RNA samples from our chronic low dose (0.25 µM of K_2_Cr_2_O_7_, 20 weeks) Cr(VI)-exposure-transformed human bronchial epithelial BEAS-2B cells and the passage-matched control cells for the m6A microarray analysis. It was found that total RNA m6A levels in Cr(VI)-transformed BEAS-2B cells were significantly higher than that in the passage-matched control BEAS-2B cells [80]. This finding was confirmed by using an ELISA-like colorimetric assay to measure the m6A RNA modification levels. Similar results were also obtained in another cell line (human bronchial epithelial cell: 16-HBE) transformed by exposure to 0.25 µM of K_2_Cr_2_O_7_ for 40 weeks. Moreover, the total RNA m6A modification levels in the lungs of mice exposed to Cr(VI) (calcium chromate) for 26 weeks were also significantly higher than that in the PBS-exposed control mouse lungs [80]. Then, we determined the mechanism of how chronic Cr(VI) exposure increased the RNA m6A modification levels. By using Western blot, we analyzed the protein levels of the writers (METTL3, METTL14, and WTAP), erasers (FTO and ALKBH5) and representative readers (YTHDF1-3). It was found that the protein levels of METTL3, but not others, were significantly higher in Cr(VI)-transformed cells than the passage-matched control cells. In addition, METTL3 protein levels were also increased in chronic Cr(VI)-exposure-caused mouse and human lung tumor tissues. Functional studies showed that stable knockdown of METTL3 in Cr(VI)-transformed cells significantly reduced their transformed phenotypes, as evidenced by the reduced capabilities in forming soft agar clones, suspension spheres, and xenograft tumors in nude mice. Furthermore, we also stably knocked down METTL3 expression in parental non-transformed cells. It was found that stable knockdown of METTL3 in parental non-transformed BEAS-2B cells significantly reduced the capability of chronic low-dose Cr(VI) exposure to induce cell transformation and cancer stem-cell-like properties [80]. Collectively, these findings provided compelling evidence showing that chronic Cr(VI) exposure causes epitranscriptomic dysregulations by increasing the expression of the m6A writer METTL3, which may play an important role in Cr(VI) carcinogenesis.

## 4. Conclusions and Future Directions

Although very limited studies have been conducted to explore the epitranscriptomic effects of exposure to metals, these pioneer studies are stimulating and offer new mechanistic insights for understanding metal toxicity and carcinogenesis. Indeed, studies revealed that the RNA m6A modification dysregulations contribute significantly to arsenic- and Cr(VI)-exposure-induced cell transformation and tumorigenesis.

Although recent studies clearly showed that exposure to metals dysregulates RNA modifications in cultured cells and in animals, further studies are needed to better define the role and mechanism of abnormal RNA modifications in metal toxicity and carcinogenesis. We propose the following areas for future studies. First, current studies on the epitranscriptomic effects of metals are largely limited to exposure to arsenic, cadmium, and hexavalent chromium, and studies in this exciting emerging field need to be extended to other toxic metals such as lead, mercury, and nickel, etc. Second, current studies focus largely on the effects of metal exposure on the RNA m6A modification, and future studies need to be extended to investigate the effects of metal exposure on other important types of RNA modifications and determine their roles in metal toxicity and carcinogenicity. Third, while current studies have provided exciting evidence showing that metal exposure caused RNA modification dysregulations, the underlying mechanisms remain largely unexplored. Further studies are needed to determine how metal exposure causes abnormal RNA modifications and how dysregulated RNA modifications mediate metal toxicity and carcinogenesis. Fourth, exposure to metals causes severe health damage. Future studies need to determine whether metal exposure dysregulated RNA modifications and their modifiers could serve as biomarkers or targets for the diagnosis and treatment of diseases resulting from metal exposure.

## Data Availability

Not applicable.

## Abbreviations

A3B	APOBEC3B
Akt	protein kinase B
ALKBH5	alkB homologue 5
AS3MT	arsenite methyltransferase
ATSDR	Agency for Toxic Substances and Disease Registry
CAT	catalase
Cr	chromium
FTO	fat mass and obesity-associated protein
HaCaT	human keratinocyte
Jak	Janus kinase
m6A	N6-methyladenosine
MAPK	Mitogen-activated protein kinase
METTL14	methyltransferase like-14
METTL3	methyltransferase like-3
MNT	MAX network transcriptional repressor
mRNA	messenger ribonucleic acid
mTOT	mammalian target of rapamycin
NAC	N-acetylcysteine
NEDD4L	NEDD4-like E3 ubiquitin protein ligase
NLRP3	NOD-like receptor protein 3
NQO1	NAD(P)H quinone dehydrogenase 1
PI3K	phosphatidylinositol-3-kinase
PTM	post-translational modification
ROS	reactive oxygen species
rRNA	ribosomal RNA
SOD	superoxide dismutase
STAT	signal transducer and activator of transcription
tRNA	transfer ribonucleic acid
UVB	ultraviolet B light (wavelength between 280–315 nm)
WTAP	Wilms’ tumor 1-associating protein
YTHDC1-2	YTH domain-containing proteins1–2
YTHDF1-3	YTH domain-containing family proteins1–3
